# Intronic microRNAs support their host genes by mediating synergistic and antagonistic regulatory effects

**DOI:** 10.1186/1471-2164-11-224

**Published:** 2010-04-06

**Authors:** Dominik Lutter, Carsten Marr, Jan Krumsiek, Elmar W Lang, Fabian J Theis

**Affiliations:** 1 Institute of Bioinformatics and Systems Biology, CMB, Helmholtz Zentrum München, Germany; 2 CIML Group, Institute of Biophysics, University of Regensburg, 93040 Regensburg, Germany; 3 Max Planck Institute for Dynamics and Self-Organisation, Bunsenstrasse 10, D-37073 Göttingen, Germany

## Abstract

**Background:**

MicroRNA-mediated control of gene expression via translational inhibition has substantial impact on cellular regulatory mechanisms. About 37% of mammalian microRNAs appear to be located within introns of protein coding genes, linking their expression to the promoter-driven regulation of the host gene. In our study we investigate this linkage towards a relationship beyond transcriptional co-regulation.

**Results:**

Using measures based on both annotation and experimental data, we show that intronic microRNAs tend to support their host genes by regulation of target gene expression with significantly correlated expression patterns. We used expression data of three differentiating cell types and compared gene expression profiles of host and target genes. Many microRNA target genes show expression patterns significantly correlated with the expressions of the microRNA host genes. By calculating functional similarities between host and predicted microRNA target genes based on GO annotations, we confirm that many microRNAs link host and target gene activity in an either synergistic or antagonistic manner.

**Conclusions:**

These two regulatory effects may result from fine tuning of target gene expression functionally related to the host or knock-down of remaining opponent target gene expression. This finding allows to extend the common practice of mapping large scale gene expression data to protein associated genes with functionality of co-expressed intronic microRNAs.

## Background

Gene regulation via microRNAs (miRNAs), small ~22 nucleotide long RNA molecules, is a strongly conserved mechanism found in nearly all multicellular organisms including animals and plants [1].

Incorporated into a protein complex mainly built of Argonaute proteins, miRNAs bind preferably to complementary regions within the 3' UTRs of mRNAs, their target sites. About 37% of the known mammalian miRNAs are located within the introns of protein coding genes, so-called host genes [2]. This has to be appreciated as a vague estimate since the number of annotated miRNAs varies strongly from 117 for *bos taurus *to 695 for *homo sapiens*, and expectations of the functionally active fraction of the genome presume amounts of miRNAs far above these numbers [3,4]. For instance, the proportions for mouse (44%) and human (53%), two of the best studied mammals, are strikingly larger. Furthermore, intronic miRNAs appear to be conserved across several species [5-7]. Although it is shown that about 26% of the mammalian intronic miRNAs may be transcribed from their own promoters [8], the majority is transcriptionally linked to their host gene expression and processed from the same primary transcript [9,10]. In human, it could also be shown that most of the intronic miRNAs show correlated expression with their host genes [11]. Besides Drosha-processed miRNAs, a second type of intronic miRNAs, termed mirtrons, is known, that bypass Drosha cleavage by splicing [12,13] but exhibit t he same co-expression patterns with their host genes. The wide occurrence of intronic miRNA raises the question whether the analysis of large-scale gene expression data principally based on protein-coding gene annotations can cope with the regulatory impact of gene expression.

Gene regulation mediated by miRNAs can be categorized into 'switch', 'tuning' and 'neutral' effects [14,15]. Switch regulation denotes a knock-down of the miRNA target. The gene product is downregulated under a specific functional threshold caused by effective translational inhibition or cleavage of the target mRNA [16]. In contrast, tuning does not inhibit target activity but tunes expression in a way such that miRNA targets are adjusted to a specific expression level required under specific cellular conditions [17]. A recent study in Drosophila shows that antropin is tuned by miR-8 to prevent neurodegeneration [18]. By neutral targets one denotes miRNA-mRNA interactions, that are functional but without any advantageous nor adverse consequences to the cell. Since the neutral regulation does not have any effect on the phenotype, it will not be discussed in this work.

**Figure 1:**
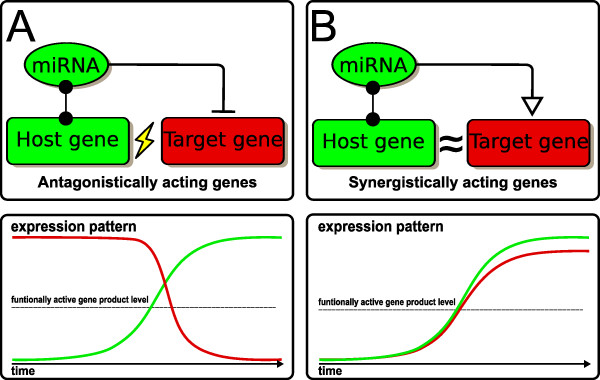
**Regulatory mechanisms**. The two proposed regulatory mechanisms of functional host to miRNA relationships. **(A) **An antagonistic effect can be achieved by miRNA-mediated downregulation of a gene with perturbing effect on a pathway or biological process regulated by the host gene. **(B) **Synergistic effect by miRNA-mediated fine tuning of a target gene with common contribution of host and target gene to a pathway or biological process. Proposed corresponding gene expression patterns are shown below the two motif figures. Genes are marked by rounded rectangles, miRNAs by ellipses. Host and intronic miRNA relations are indicated by an edge with a dot. MiRNA target tuning regulation is indicated by a blank triangle, inhibition is indicated with a stop.

It is a common paradigm in biology that conservation on DNA sequence level also implies a conservation of function. Therefore we hypothesize that the widespread appearance of the transcriptional junction of a protein coding gene and the regulatory miRNA implies a common function. Specifically, the co-regulation of a miRNA with its host gene may include two different main functions: (i) An antagonistic effect is achieved by miRNA-mediated knock-down of genes with perturbing effects on a pathway or biological process activated by the host gene. The combined expression of an effector gene and a miRNA, which blocks translation of such antagonistic gene products, is a simple but elegant way to promote and support host gene functionality (Figure 1A). (ii) A synergistic effect can be achieved by adjusting the protein expression levels of intronic miRNA targets towards intended optimal concentrations. A specific ratio between host and target gene products then allows for effective and optimized cooperative actions of co-regulated genes (Figure 1B). In this work we assume that the proposed antagonistic effect is mainly mediated by switch regulation, whereas tuning of targets mainly mediates synergistic effects.

Genes sharing a common function, such as being involved in the same biological pathway, tend to share similar regulatory mechanisms and therefore appear as co-expressed genes in their expression profiles [19]. Thus, genes with correlated time-dependent expression patterns are likely to be involved in functionally related cellular processes. At least it is very unlikely that co-expressed genes act in an antagonistic manner. In contrast, anti-correlated expression patterns would promote the assumption that the participating genes take part in either unrelated or antagonistic processes. Furthermore, there is increasing evidence that many miRNAs cause degradation of their targets [20-22], which referring to mRNA expression will appear as anti-correlated expression patterns. In human, a functional relation between the host gene *GRID1 *and the intronic miR-346 has been shown recently [23] and the hereby proposed antagonistic effect has been proven for the intronic miR-338 and its host gene *AATK *[24]. Furthermore, a recent study has shown that the intronic miR-208a, expressed with its murine heart-specific host gene *Myh6*, negatively regulates two proposed targets, namely thyroid hormone-associated protein 1 and myostatin, both negative regulators of muscle growth and hypertrophy [25]. However, these findings prove the proposed effects only for single miRNAs. To ensure that these findings were not individual cases, but also generally detectable we applied several statistical methods on large scale data.

In this work, we investigated the functional relation between miRNA host genes and putative targets of corresponding intronic miRNAs with a data-driven approach based on large-scale gene expression data and a knowledge-based approach using gene annotations. We analyzed large scale gene expression profiles which are widely available and provide a basis to reveal gene expression phenotypic models. Furthermore, functional gene annotations as provided by the Gene Ontology (GO) [26] give information about a common or strongly related function of two genes, for instance host and target. We hypothesize that functional relations between miRNA host genes and related target genes appear in significantly correlated expression patterns. Furthermore, we expect that host and target gene sets are closer related in the GO as randomly sampled sets, for both antagonistic and synergistic motifs as introduced in Figure 1.

## Results and Discussion

### Targets of similarly expressed host genes show correlated expression patterns

We studied the relationship between host and target genes in three different mouse developmental microarray datasets (see methods): embryonic stem cell development (SCD) [27], somitogenesis (SG) [28] and neurite outgrowth (NO) [29]. We chose developmental datasets since regulatory effects of miRNAs are known to be strongly present in developmental processes [30]. During cell differentiation, groups of genes driving specific developmental processes are often commonly regulated, resulting in similar expression patterns in time course-data. A synergistic relationship between host and the miRNA target genes of differentiating cells is then indicated by positively correlated gene expression patterns. In reverse, antagonistic processes are expected to show anti-correlated or uncorrelated expression patterns between host and related target genes.

Since we argue that correlated expression indicates potentially common host gene functions, we initially tested for correlations between the expression patterns of known host genes. In order to generate statistically robust results (independent of data and prediction errors) we did not analyze single gene expression patterns but argue on groups of correlated genes. Therefore, for each dataset we identified all miRNA host genes and clustered their time-courses according to correlations above 0.8 (see methods).

Within all analyzed cell differentiation datasets, host genes tend to be co-expressed in clusters. As a result of our clustering we obtained seven host gene clusters with more than 5 host genes from all three datasets (see Table 1).

**Table 1:** Host gene cluster size and number of target genes, predicted using TargetScan (TS).

		all predicted		positively correlated		negatively correlated	
	Hosts	targets	score	targets	score	targets	score
SCD I	9	282	1.78	141	1.76	133	1.80
SCD II	8	778	2.19	382	2.27	388	2.11
SG I	13	1531	1.84	793	1.82	727	1.86
SG II	21	1971	1.85	1013	1.90	942	1.80
SG III	7	621	1.99	333	2.02	283	1.95
NO I	10	873	1.81	351	1.81	512	1.80
NO II	17	1286	2.19	706	2.22	567	2.16

For each cluster we calculated the similarity score between host and all predicted target genes. Additionally, target genes were split up into positively and negatively correlated targets and similarity scores related to the host genes were calculated. The p-values determined by a comparison of functional GO similarities between host and predicted targets to randomly chosen sets of target genes of identical size were all significant after Bonferroni correction (all clusters *p *< 10^-5^). Independent from the sign of correlation, we determined a significantly higher similarity than expected for all analyzed clusters.

Intriguingly, some host genes appear to be clustered preferentially across the experiments. The genes *H19, Igf2, Lpp, Plod3*, and *Rnf130 *were clustered in the two clusters SCD I and NO I, and the genes *Chm, Copz1, Dnm1, Nupl1*, and *Sf3a3 *in the clusters SG I and NO II.

For each host gene cluster we identified the intronic miRNAs and all their expressed targets. Most prediction tools for miRNA target site prediction vary qualitatively and quantitatively. In order to get more confident predictions, we used a consensus model (CM) of several miRNA target prediction tools (see methods) [31]. Detailed lists of all analyzed miRNAs/clusters in this work including host genes and loci, are available as additional files 1 and 2.

**Figure 2:**
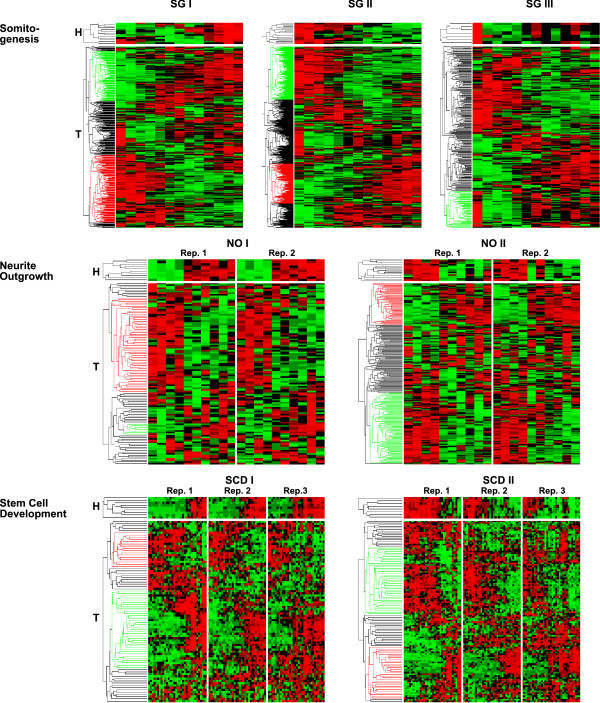
**Clustered heat maps**. Clustered heat maps for the seven host gene clusters (**H**) and the corresponding target gene expression profiles (**T**). For all three time course datasets only clusters with more than five host genes are shown. Each row corresponds to one gene expression pattern, each column to a measurement. Time dependent measurements are shown in ascending order from left to right. The expression level of each gene is standardized so that the mean is set to 0 and the standard deviation is 1. Expression levels above and below 0 are color-coded: red indicates for high and green for low expression levels, respectively; black for zero expression values. Biological replicates of the three datasets are in order from Rep. 1 to Rep. 2 and Rep. 3, respectively. Colored subtrees in the dendrogramm derived from hierarchical clustering denote for co-expressed (green) or anti-correlated (red) gene expression of predicted targets. (**Somitogenesis**) The dataset splits up into three host gene clusters, SG I with 13, SG II with 21, and SG III with 7 host genes. (**Neurite Outgrowth**) Two cluster with 10 (NO I) and 17 (NO II) host genes could be identified with similar behavior of host and target genes in both replicates. (**Stem Cell Development**) Two host gene clusters containing 9 (SCD I) and 8 (SCD II) host genes were identified. All host and target genes show similar behavior in all three replicates. For each dataset, flipped expression patterns between the host/target clusters are striking (SG I vs. SG II; NO I vs. NO II; SCD I vs. SCD III).

We performed a hierarchical cluster analysis for the seven clusters based on the expression data of the target genes (see Figure 2). All resulting trees mainly split up in two subclusters: one subcluster of genes with positively correlated expression patterns and one with anti-correlated expressions compared to the host genes. Furthermore, within each dataset, the resulting trees of at least two target gene groups appeared to show completely flipped expression patterns of the main subclusters (SG I vs. SG II; NO I vs. NO II; SCD I vs. SCD III).

These results fit well to the observation that miRNAs dampen the output of preexisting mRNAs or optimize required protein output as it is proposed for metazoans [32]. Additionally, it was shown that genes preferentially expressed at the same time and place as a miRNA tend to avoid sites matching the miRNA [33]. In contrast, co-expression of transcripts with evolutionary conserved miRNA binding sites would then arise from a functional requirement.

The clear discrimination between the two expression patterns suggests a gradual order of differentiating cells, whereas miRNAs function as enhancers of robustness in gene regulation [34,35]. A plausible explanation would be that shortly after initiation of the differentiation process, genes that arrange the differentiating cell towards its new function are up-regulated. In this stage miRNAs are activated to inhibit processes required for self-renewal of stem cells but act perturbing during differentiation. After this reorganization the cell adopts its new functions. In this phase genes are up-regulated which now fulfill the cell's new responsibilities and simultaneously block activity that was only required for differentiation.

### MiRNA host gene clusters and related target genes show significant correlations of their expression patterns and functional similarities

In order to confirm the above observed we statistically compared gene expression patterns of host genes with the expression patterns of predicted target genes and sets of randomly sampled genes. For each cluster, we determined the correlations between all hosts and predicted targets and all hosts and 500 sets of randomly sampled genes. Using Wilcoxon's rank sum test we tested the observed correlations to result from distributions with equal median (see methods).

To avoid bias in our CM, we further used three independent miRNA target prediction tools, namely Pictar (PT) [36], TargetScan (TS) [37] and RNA22 (R) [38]. For each host gene cluster and each single host gene, expression patterns were compared to the expression of predicted targets. Only clusters with predicted and expressed targets in the respective dataset were used in the following analysis.

**Figure 3:**
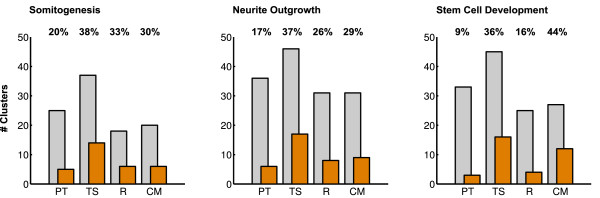
**Results: expression analysis**. Results of the host gene cluster based expression analysis. Grey bars denote the number of all identified host gene clusters including unclustered hosts with expressed target genes, predicted by Pictar (PT), TargetScan (TS), RNA22 (R) and our consensus model (CM). Orange bars denote the number of clusters with significantly correlated target gene expression patterns. The relative fraction of significant clusters for each dataset and miRNA target prediction tool is denoted.

Concordant for all used methods and all analyzed datasets, we determined that 9 to 44% of the identified host gene clusters were significantly positively correlated or anti-correlated to their target gene expressions (see Figure 3). Thereby, the observed correlations between hosts and targets were independent from the analyzed dataset. The identification of significantly correlated target gene expressions based on the four used target prediction methods appears to be relatively similar between the datasets. The average amount of host gene clusters with significantly correlated target expression for the three datasets varies between 26% and 30%.

Comparing the four tools, PT performs strikingly weaker (15%) than TS, R and the CM with regard to the mean fraction of host gene clusters with significantly correlated predicted target expressions (37%, 25% and 34%). Since the number of targets predicted with PT for each host gene is on average considerably smaller compared to the two other methods, false positive predictions have a larger effect on the determined p-values.

Although, the CM graph is less dense than the other graphs as well as notably smaller than the TS and R graph, it performs best in this analysis with an equal fraction of significantly regulated clusters. However, our results are consistent over all datasets and all different miRNA target site prediction tools.

To further validate our results we used miRNA-mRNA interactions identified by Argonaute HITS-CLIP in the mouse brain [39]. From the 20 miRNAs tested in this study two (miR-153, miR-708) were identified as intronic. After mapping these to our expression data we found the miR-708 host gene *Odz4 *expressed in the NO and SG datasets. From the 374 targets, 209 targets were expressed in the NO dataset. After clustering these target genes, the resulting tree again splits up into two main subclusters, one with positively correlated and one with anti-correlated gene expression profiles (Additional file 3 - Figure A).

The histogram over the correlation coefficients also confirms an equal distribution of positive and negative correlation coefficients (Additional file 3 - Figure B). The considerable fraction of only weakly correlated target genes may result from (i) false positive experimental miRNA-mRNA associations, (ii) neutral regulatory effects, or (iii) experimental noise.

### Functional relation between host and target genes includes synergistic as well as antagonistic effects

The results so far indicate a non-directed functional relation between host genes and intronic miRNAs, but do not provide any information on positive or negative correlations. To get robust results, we only used the two tools with the highest number of overall identified host-target gene clusters, which were TS and the CM for the following analysis.

**Figure 4:**
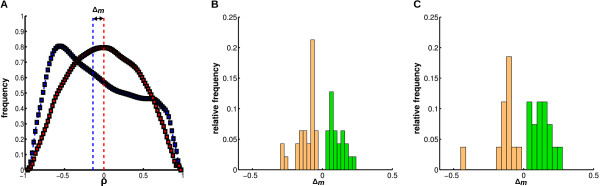
**Results: pattern correlations**. Comparison of the expression pattern correlations. (**A**) Shown are the distributions of correlation coefficients *ρ *between host and target gene expression patterns (blue) of Cluster NO I and correlation coefficients *ρ *between the same host genes and sampled target genes (red). The medians are illustrated by blue and red lines, respectively. Δ_*m *_indicates the difference between the two medians. A missing relation between host and target gene expression would result in Δ_*m *_= 0. The distributions of Δ_*m *_taken over all significant clusters of the three datasets are shown in the two histograms for TargetScan (**B**) and our consensus model (**C**). Missing distances of Δ_*m *_= 0 in both histograms indicate that all significant clusters deviate from the null model (sampled data). Both histograms show distributions with two maxima, indicating that positive (green) and negative (orange) correlations are approximately equally distributed over all analyzed clusters.

To test whether one or both of the two proposed functional effects - synergistic or antagonistic - may be identified in our data, we calculated the distance between the medians, derived from the correlation distributions between host and predicted target genes and hosts and randomly sampled targets (Figure 4A and methods). The resulting distances Δ_*m *_combined from all three datasets can be seen in Figure 4B and 4C. Both distance distributions show a bimodal distribution with a local minimum at Δ_*m *_= 0, but no significant shift towards a negative or positive correlation. Hence, based on the assumption that highly positive or negative correlation of gene expression patterns indicates similar or opposite functions, we infer that the proposed synergistic and antagonistic effects appear to be equally represented in our data.

Since our investigation is only based on mRNA expression data and further information on protein levels is missing, the real impact on translation stays obscure in this analysis. However, it could be shown that most of miRNA-mRNA interactions function as fine-tuning adjustments to the proteome [40]. Considering the fact that our experimental analysis was based on mRNA expression data, only knock-down effects mediated by target cleavage are directly visible. However, in agreement with previous work [40,41], the massive appearance of positively correlated miRNA and target expressions strongly indicates tuning effects of varying translational repression. Furthermore, as our findings so far were derived from developmental data, we have to state that the observed effects could be related to these datasets only.

### Host and target gene sets display enriched functional similarity

The significantly correlated expression patterns between host genes and miRNA target genes support the notion that intronic miRNA regulation improves host-associated biological functions by either tuning or dampening the expression of target genes. We assume that this relation is also apparent via shared functional annotations. To test this hypothesis, we determined the commonly used functional similarity of gene products based on Gene Ontology (GO) [42] between a single or multiple host genes and their set of target genes. We then calculated the significance of the mean functional similarity by comparing the target set with randomly sampled sets of miRNA target genes (see methods).

We analyzed the previously defined clusters SCD I - NO II and calculated mean functional similarities between the host and all TS predicted target genes. According to the proposed synergistic and antagonistic effects, we divided the target genes upon their expression pattern correlations with the host genes into two groups of positive and negative correlated targets and calculated similarity scores. Results are shown in Table 1. All host gene clusters display a significantly higher functional similarity to their predicted target genes as compared to the null model of randomly chosen target genes (*p *< 0.01, after Bonferroni-correction). Comparing the similarity scores derived from the positively and negatively correlated target genes showed no significant differences between the two distributions. Independent from the sign of correlation, we determined a significantly higher similarity score than expected for all analyzed clusters.

**Figure 5:**
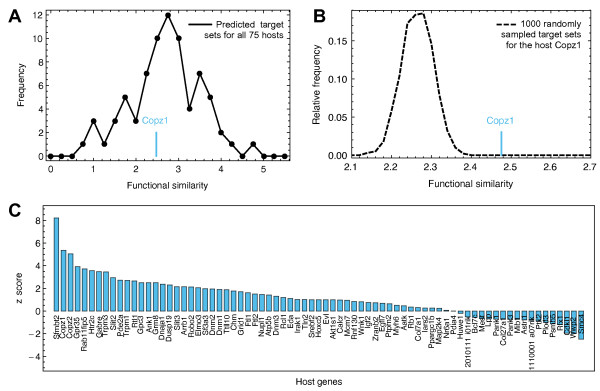
**Results: Functional similarity**. Functional similarity of host and target gene sets as predicted by TargetScan. (**A**) Frequency distribution of the functional similarity score for all 75 host-target relations. For each single host gene and its set of target genes, we calculate a mean score based on the GO annotation 'biological process'. The mean functional similarity of the host gene *Copz1 *to its predicted targets is 2.48 (blue line). (**B**) Comparison of the real functional similarity score the host gene *Copz1 *with a null model distribution. For the null model, a random set of miRNA target genes of the same size has been chosen 1000 times and the functional similarity score has been calculated. The real score of *Copz1 *deviates significantly from the null model distribution, resulting in a high *Z*-score. (**C**) *Z*-scores for all annotated host genes. A total of 21 out of 75 host genes show *Z*-scores > 2 and thus display a significantly higher functional similarity as expected from a random sample of target genes.

To check whether a high functional similarity can be found for all host-target relations independent of expression patterns, we additionally calculated the functional similarity score for all single host genes and their predicted target gene sets. We expected the most robust results for the largest network of predicted miRNA target gene associations, since the score is given by the mean of all host gene - target gene pairs. In Figure 5A, we plotted the frequency distribution of similarity scores for TS. We found that the scores are well distributed within the range of 0 and 5. We compared each similarity score with a null model, where the same number of target genes is randomly selected from all miRNA target genes as provided by TS. For the host gene *Copz1*, for example, we found a significantly larger functional similarity to its targets as compared to 1000 randomly selected sets of miRNA targets (see Figure 5B).

For all annotated host genes with available annotations for the respective targets, we calculated p-values and *Z*-scores as measures of deviation from the null model. We found that surprisingly many host-target relations deviated from the null model, with high *Z*-scores as can be seen in Figure 5C. As many as 57 of all 75 host genes annotated in the ontology 'biological process' exhibited a greater similarity to their targets (*z *> 0) than expected by chance, 30 of them with a p-value < 0.05. For those pairs of host and target genes, a strong correlation in terms of their annotated 'biological process' existed. For the other prediction tools used on in this study, a similar trend to high *Z*-scores could be observed (Additional file 3 - Figure C). However, these predictions comprise less annotated host genes (48 and 45 for PT and CM, respectively) and also about 10 times less links, rendering significant deviations less possible (see methods for details).

With the use of GO annotations we could show that intronic miRNAs tend to target genes that are functionally more similar to the host genes than randomly chosen genes. The strong bias towards positive *Z*-scores and the absence of significant dissimilarities between host and target genes, independent from the sign of correlation, agrees with both former proposed regulatory principles (Figure 1A, B). Notably, GO terms are not classified with respect to antagonistic or synergistic effects but on biological relations. For instance, two pathways with conicting regulation on a cellular process like 'cell growth' are both children of the parental term and therefore close within the GO tree. Furthermore, two genes can have opposing regulatory effects on one pathway and would be still grouped together in the same term.

## Conclusions

The results of this work show that the genomic linkage between intronic miRNAs and their host genes coincides with a functional relation. Using a data-driven as well as a knowledge-based approach, miRNA host genes and related target genes were analyzed towards functional relations. Expression patterns were obtained from three developmental datasets. Correlated expressions of host and miRNA target genes deviated significantly from a random model. Both, positive and negative correlation patterns have been observed in approximately equal amounts. A further GO analysis of the predicted miRNA-mRNA interaction network confirmed that host and predicted target genes tend to be annotated with similar or related terms, compared to a random model. Taken together, our results indicate either synergistic or antagonistic regulatory effects mediated by either downregulation of genes with an opposed function or fine-tuning of miRNA targets, co-operative to the host gene. This finding extends the common perception of gene expression analysis with a new regulatory functionality.

## Methods

### Microarray data and preprocessing

All analyzed datasets were taken from the GEO [43] database: (i) The stem cell development (SCD) dataset consists of three cell lines (R1, J1, V6.5) differentiated into embryoid bodies (EB) at 11 time points from t = 0 h until t = d 14. From each time point and each cell line 3 technical replicates were measured (combination of three cell line differentiations GSE2972, GSE3749, GSE3231). (ii) Within the somitogenesis dataset (SG) gene expression was measured from synchronized C2C12 myoblasts at 13 timepoints from t = 0 h until t = 6 h (GSE7012). (iii) The neurite outgrowth (NO) and regeneration dataset consists of transcriptional activity, measured from dorsal root ganglia during a time course of neurite outgrowth in vitro under two conditions: untreated and under potent inhibitory cue Semaphorin3A. Measurements were taken at 5 time points from t = 2 h until t = 40 h including two technical replicates (GSE9738).

Affymetrix raw data were preprocessed using Bioconductor's R package *simpleaffy *[44]. Data was normalized and detection calls were determined. Expression values were calculated using the RMA algorithm. Each dataset was filtered independently to remove all probesets with an absent flag in more than two third of all datapoints within the whole experimental setup.

Gene names and gene symbols for each probeset were derived from the Bioconductor Affymetrix Mouse Expression Set 430 annotation data package (moe430a.db). Gene symbols represented by more than one probeset were set to the median expression values.

### Expression profile based analysis

Host gene clusters were defined upon a correlation-based adjacency matrix. For each microarray dataset we selected all known miRNA host genes and calculated a correlation matrix based on their expression profiles. Each entry representing a correlation coefficient above 0.8, was set to 1, all others to 0. This adjacency matrix now forms a graph of host genes. A host gene cluster was then defined as a maximal connected subgraph of this graph. This equals nearest neighbor method applied to hierarchical clustering algorithm with a defined cutoff of 0.8 of the dendrogramm. For each host gene cluster containing *M *host genes, the *N *corresponding target genes were determined upon the three miRNA target prediction tools. We calculated the cluster specific miRNA degree *d*_*i *_= #*T*_*i*_/#*H*_*i *_where #*T*_*i *_is the number of target genes and #*H*_*i *_is the number of host genes of cluster *i*.

Depending on the respective expression profiles, we calculated the *M *× *N *cross-correlation coefficients between all hosts and all targets. As a null model we randomly sampled *N *targets 500 times. For each sample we calculated all *M *× *N *correlations. Statistically significant differences between the correlation distributions of our clusters and sampled data were estimated by determining p-values using Wilcoxon's rank sum test.

Distances between the medians of the correlation distributions were calculated as(1)

with *C*_*c *_being the correlation distribution between the host and the target genes of one cluster and *C*_*s *_being the correlation distribution between host genes and sampled target genes. Hierarchical cluster analysis was performed using Matlab's Bioinformatics toolbox http://www.mathworks.com using average linkage with Euclidean distance metric.

### Intronic miRNAs and target prediction

A list of all murine intronic miRNAs and their host genes was downloaded from the miRBase website http://microrna.sanger.ac.uk. PT predictions were download from the UCSC genome browser http://genome.ucsc.edu. TS conserved miRNA target site predictions were downloaded from the TS website http://www.targetscan.org. In contrast to PT and TS, R is a prediction tool that does not rely on cross-species conservation. The data was downloaded from their website http://cbcsrv.watson.ibm.com/rna22.html. Redundant gene-to-miRNA relationships were removed from all datasets.

The CM prediction graph used in our analysis was built of five different miRNA target site prediction tools. Additionally to PT and TS we used predictions from PITA [45], Miranda http://www.microrna.org[46], and TargetSpy http://www.targetspy.org[47]. From all predictions based on RefSeq transcript IDs, we filtered out only miRNA-transcript relations that were predicted by a minimum of four different tools. Transcript mapping to gene symbols was done using a local copy of the RefSeq database (September 2008) [48].

**Figure 6:**
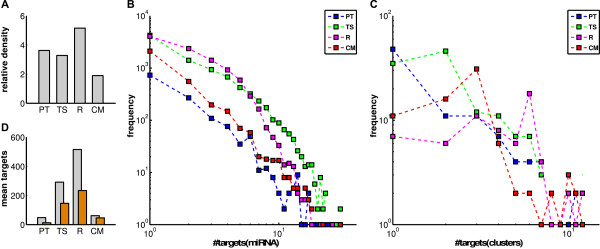
**Graph properties**. Properties of the four miRNA-target bipartite graphs. (**A**) The relative densities, number of existing edges divided by all possible edges, in percent of the four graphs for Pictar (PT), TargetScan (TS), RNA22 (R) and consensus model (CM). (**B**) Log-log plot of the number of predicted miRNA targets for all four different prediction graphs. (**C**) Log-log plot of cluster specific miRNA target recovery for all four different prediction graphs (for details see text). (**D**) The mean of the numbers of predicted miRNA targets of the complete graphs (grey), and cluster-specific recovery of miRNA targets (orange): Mean of the sums of all identified targets of one host gene cluster divided by the sums of all host genes of the cluster.

These genome-wide predictions can be represented by a bipartite graph, where the two different sets of nodes represent the miRNAs and the target genes, respectively, and the predicted interactions are formed by the edges. The three graphs vary primarily in their absolute sizes. PT with 242 miRNAs and 1335 overall predicted targets is very small compared to TS (382 miRNAs, 8879 targets), R (233 miRNAs, 9997 targets) and CM (219 miRNAs, 3249 targets). In Figure 6A relative densities for all graphs and in Figure 6B all degree distributions are shown. For each cluster a mean miRNA target recovery was calculated as the fraction of the number of all predicted and recovered target genes of one cluster to the number of clustered host genes. These distributions again are strikingly similar whereas the mean still varies strongly (Figure 6C, D).

The fraction of the cluster-specific miRNA degree compared to the complete graph miRNA degree of CM is very high (76%) compared to the other methods (TS: 50%, PT: 27%). Since TS predicts the highest number of targets per miRNA, one also expects a relatively large recovery of target genes within the dataset. The PT graph is the densest graph of all but also the smallest one, hence the weak recovery of targets. One reason for the high target recovery of the CM might be that the used prediction tools for the CM score are all trained upon validated data. Therefore, the resulting miRNA-target predictions contain more training data as the PT and TS, which results in the high recovery rate.

### Functional similarity of host genes and target gene sets

We assume that host genes confer regulatory control by translational inhibition of the respective intronic miRNA target genes in possibly related biological processes. To test this hypothesis for all hosts and target genes, we compare the similarity of their respective annotations. Functional gene annotations as provided by the GO [26] classify genes according to their function, associated biological processes or appearance within defined cellular components. They are organized hierarchically, typically in a directed acyclic graph. To each gene more than one classification term can be assigned.

The functional similarity between a host and a target was defined by *Resnik's measure *as described in [42] and calculated using the ProCope software suite [49]. This method scores relationships between genes by common appearance within one or more terms or, more abstract, by analyzing their distance within the GO graph. For genes with multiple term annotations the maximum scoring GO term pair was used. The functional similarity between a host and a set of targets was determined as the mean of all single host-target scores. For our study, we downloaded the most recent GO files and mouse gene annotation lists from the GO website (January, 2009).

In order to assign statistical significance of the functional host-target similarities in our network, we compared the average similarity of each host to all of its targets against 100.000 randomized networks. To evaluate the host-cluster to target relations, we compared the average host-target similarities in the real network against 100.000 networks with randomized target sets for each host cluster. We calculated a p-value as the relative number of random samples with scores exceeding the score from the data sample. The *Z*-score was calculated as the deviation of the real score s from the mean *m *of the sampled distribution, divided by its standard deviation *σ*, .

## Authors' contributions

DL planned and performed the study and prepared the original draft. CM performed the functional similarity part of the analysis which was implemented by JK. EL guided the gene expression analysis and FT coordinated the study. All authors read and approved the final manuscript.

## Supplementary Material

Additional file 1 I**ntragenic microRNAs**. Table of all inragenic miRNAs and corresponding host genes used in this analysis. The table lists the miRNA name, gene symbol and miRNA locus in tab delimited columns. Click here for file

Additional file 2 **host gene cluster**. Table of all identified host gene clusters. Each row lists one host gene cluster with cluster identifier and the list of comma separated host gene symbols. The cluster identifier is composed of the dataset identifier as used in the manuscript and numbering in Roman numbers. Click here for file

Additional file 3 **Supplementary Figures A, B, and C**. (A) Heatmap of the Ago ternary map based *Odz4 *target genes and the corresponding Correlation Coefficients (B). (C) *Z*-scores for all annotated host genes based on the Pictar and consensus model target gene predictions. Click here for file
