# Supplementary material for: Intronic microRNAs support their host genes by mediating synergistic and antagonistic regulatory effects
Source: BMC Genomics. 2010 Apr 6;11:224. doi: 10.1186/1471-2164-11-224 (PMC2865499; doi:10.1186/1471-2164-11-224)
Supplement: Additional file 3 — Supplementary Figures A, B, and C. (A) Heatmap of the Ago ternary map based Odz4 target genes and the corresponding Correlation Coefficients (B). (C) Z-scores for all annotated host genes based on the Pictar and consensus model target gene predictions. [file 1471-2164-11-224-S3.PDF]

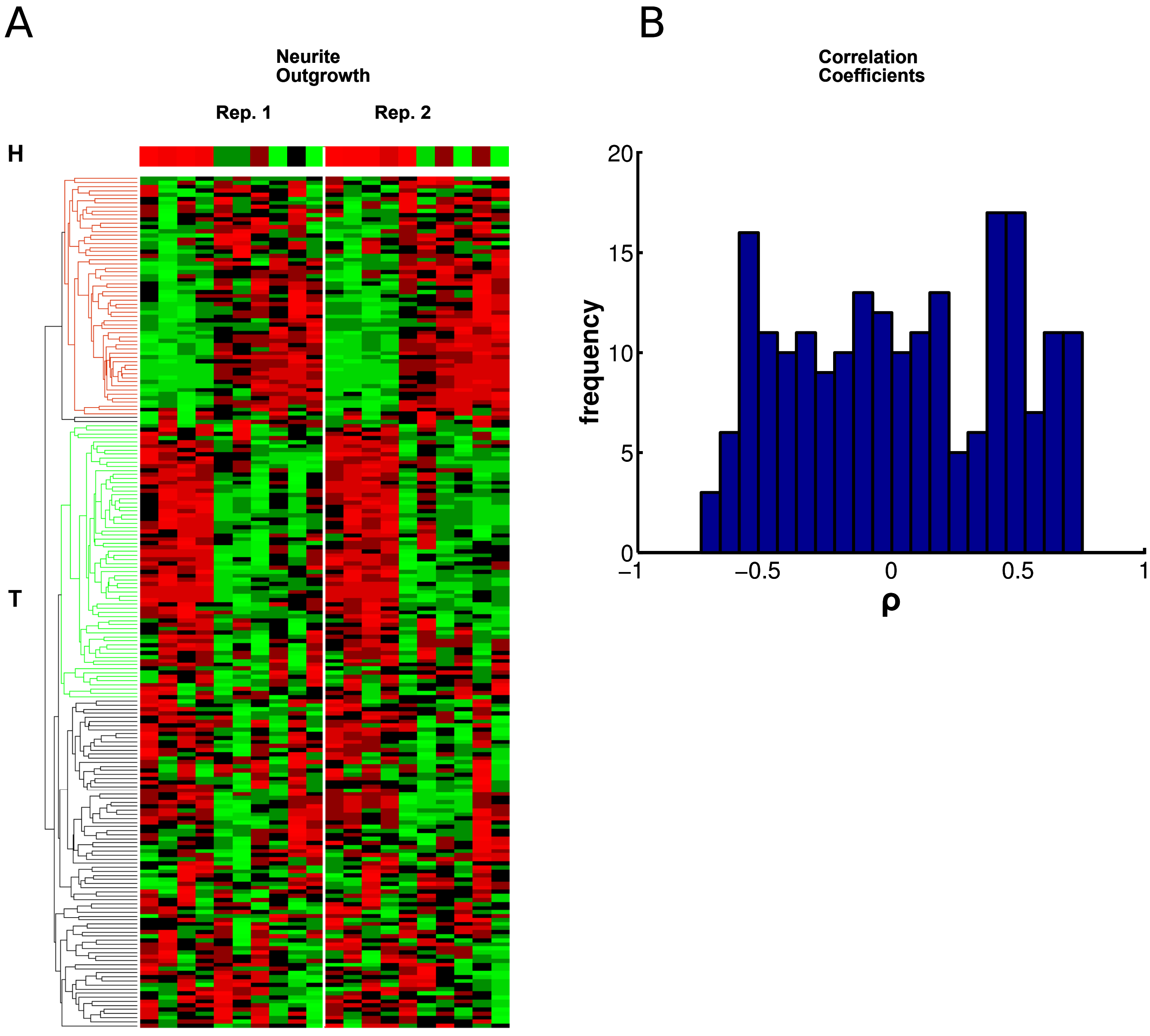

(**A**) Clustered heat map for the host gene *Odz4* (**H**) and the expression profiles of the target genes (**T**) taken from the Ago ternary map available at `http://ago.rockefeller.edu/`. Expression Values were taken from the Neurite Outgrowth dataset. Each row corresponds to one gene expression pattern, each column to a measurement. Time-dependent measurements are shown in ascending order from left to right. The expression level of each gene is standardized so that the mean is set to 0 and the standard deviation is 1. Expression levels above and below 0 are color-coded; red indicated for high and green for low expression levels, respectively; black for zero expression values. Colored subtrees in the dendrogramm derived from hierarchical clustering denote for co-expressed (green) or anti-correlated (red) gene expression of predicted targets.

(**B**) The histogram shows the corresponding correlation ρ coefficients between the expression profile of the host gene *Odz4* and the target gene profiles shown in figure A.

C

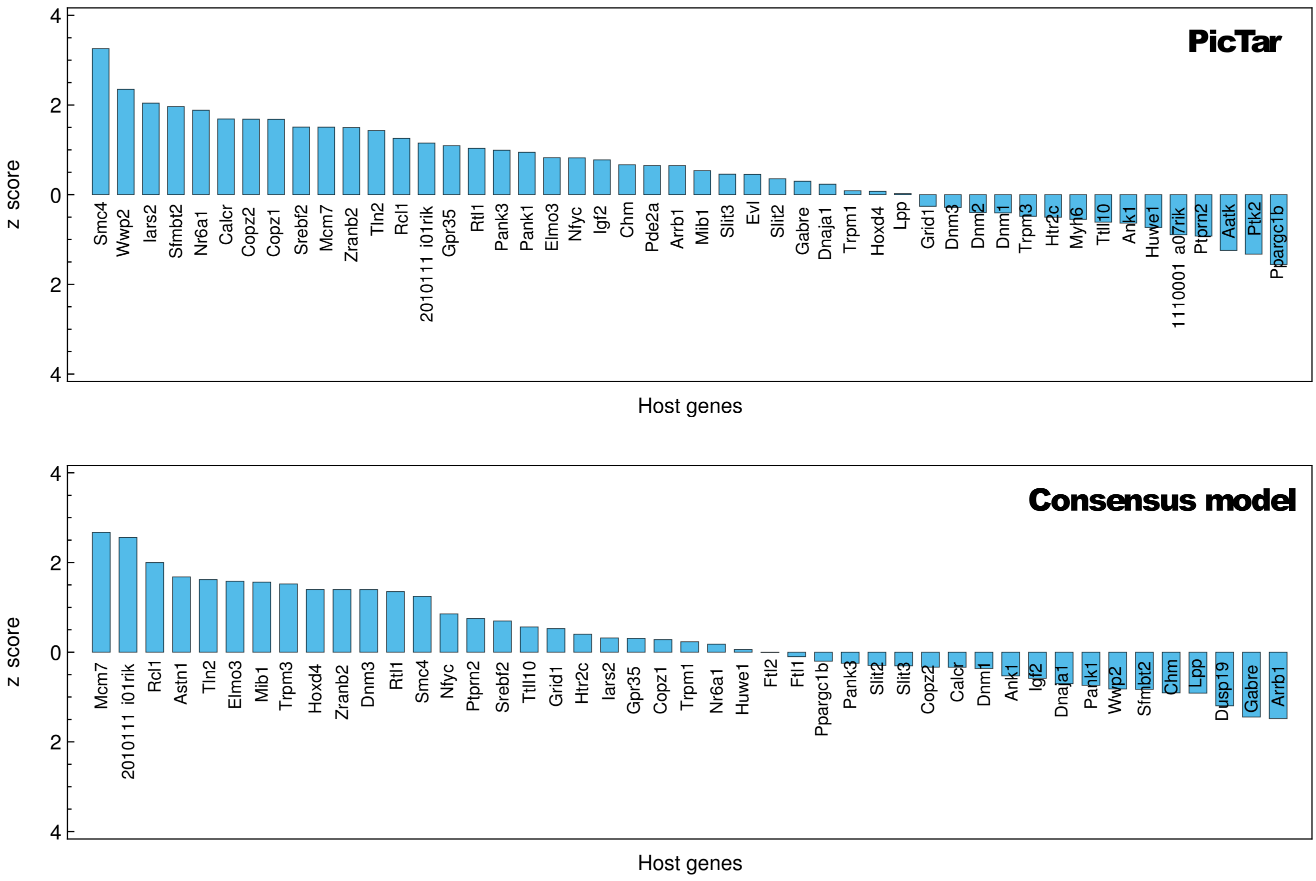
PicTar
z score
4
2
0
2
4
Smc4
Wwp2
Iars2
Sfmbt2
Nr6a1
Calcr
Copz2
Copz1
Srebf2
Mcm7
Zranb2
Tln2
Rcl1
2010111 i01rik
Gpr35
Rtl1
Pank3
Pank1
Elmo3
Nfyc
Igf2
Chm
Pde2a
Arrb1
Mib1
Slit3
Evl
Slit2
Gabre
Dnaja1
Trpm1
Hoxd4
Lpp
Grid1
Dnm3
Dnm2
Dnm1
Trpm3
Htr2c
Myh6
Ttll10
Ank1
Huwe1
1110001 a07rik
Ptprn2
Aatk
Ptk2
Ppargc1b
Host genes
Consensus model
z score
4
2
0
2
4
Mcm7
2010111 i01rik
Rcl1
Astn1
Tln2
Elmo3
Mib1
Trpm3
Hoxd4
Zranb2
Dnm3
Rtl1
Smc4
Nfyc
Ptprn2
Srebf2
Ttll10
Grid1
Htr2c
Iars2
Gpr35
Copz1
Trpm1
Nr6a1
Huwe1
Ftl2
Ftl1
Ppargc1b
Pank3
Slit2
Slit3
Copz2
Calcr
Dnm1
Ank1
Igf2
Dnaja1
Pank1
Wwp2
Sfmbt2
Chm
Lpp
Dusp19
Gabre
Arrb1
Host genes
